# The Core- and Pan-Genomic Analyses of the Genus *Comamonas*: From Environmental Adaptation to Potential Virulence

**DOI:** 10.3389/fmicb.2018.03096

**Published:** 2018-12-12

**Authors:** Yichao Wu, Norazean Zaiden, Bin Cao

**Affiliations:** ^1^State Key Laboratory of Agricultural Microbiology, College of Resources and Environment, Huazhong Agricultural University, Wuhan, China; ^2^Singapore Centre for Environmental Life Sciences Engineering, Nanyang Technological University, Singapore, Singapore; ^3^School of Civil and Environmental Engineering, Nanyang Technological University, Singapore, Singapore

**Keywords:** *Comamonas*, metabolic system, biofilm, virulence, nitrate reduction

## Abstract

*Comamonas* is often reported to be one of the major members of microbial communities in various natural and engineered environments. Versatile catabolic capabilities of *Comamonas* have been studied extensively in the last decade. In contrast, little is known about the ecological roles and adaptation of *Comamonas* to different environments as well as the virulence of potentially pathogenic *Comamonas* strains. In this study, we provide genomic insights into the potential ecological roles and virulence of *Comamonas* by analysing the entire gene set (pangenome) and the genes present in all genomes (core genome) using 34 genomes of 11 different *Comamonas* species. The analyses revealed that the metabolic pathways enabling *Comamonas* to acquire energy from various nutrient sources are well conserved. Genes for denitrification and ammonification are abundant in *Comamonas*, suggesting that *Comamonas* plays an important role in the nitrogen biogeochemical cycle. They also encode sophisticated redox sensory systems and diverse c-di-GMP controlling systems, allowing them to be able to effectively adjust their biofilm lifestyle to changing environments. The virulence factors in *Comamonas* were found to be highly species-specific. The conserved strategies used by potentially pathogenic *Comamonas* for surface adherence, motility control, nutrient acquisition and stress tolerance were also revealed.

## Introduction

The *Comamonas* genus belongs to the Burkholderiales order in the Betaproteobacteria class. They are a group of Gram-negative, non-fermentative and rod-shaped bacteria (Willems and Vos, 2006). While most members of this genus are aerobic chemoheterotrophs, some of them, including *C. nitrativorans, C. koreensis*, and *C. denitrificans*, are facultative anaerobes capable of using nitrate or ferric iron (Fe^3+^) as an alternative electron acceptor (Gumaelius et al., 2001; Wu et al., 2009). The *Comamonas* genus has been reported as one of the major members of microbial communities in various natural and engineered environments (Supplementary Table S1; Auguet et al., 2015; Guo et al., 2015; Sotres et al., 2016). Although *Comamonas* spp. are considered as non-pathogenic or rare opportunistic pathogens to human, some *Comamonas* species have been suggested to be involved in some invasive infections, like appendicitis, bacteraemia and meningitis (Tsui et al., 2011; Opota et al., 2014; Zhou et al., 2018). Several cases of *Comamonas*-associated infection, in particular, infections caused by *C. testosteroni, C. kerstersii*, and *C. aquatica* have been reported in recent years (Farshad et al., 2012; Almuzara et al., 2013; Orsini et al., 2014).

The versatile catabolic capabilities of *Comamonas* have been studied extensively in the last decade (Boon et al., 2000; Wu et al., 2006; Liu et al., 2007). They have been shown to be capable of catabolizing a wide range of organic substrates, including amino acids, carboxylic acids, steroids and aromatic compounds. In contrast, little is known about the ecological roles and adaptation of *Comamonas* to different environments as well as the virulence of potentially pathogenic *Comamonas* strains.

The development of genome sequencing provides a great opportunity to describe *Comamonas* genomic traits at the genus level. The objective of this study was to elucidate the core- and pan-genomic feature of the *Comamonas* genus which sheds light on their potential ecological role in different habitats, including natural and engineered environments as well as medical settings. Virulence factors of the potentially pathogenic strains were also identified. Specifically, we analyzed the entire gene set (pangenome) of the *Comamonas* genus using all the available genome sequences. The genetic distribution was revealed and the conserved gene set (core genome) across all the genomes was identified. To investigate their ecological functionality, the key metabolic features, signaling systems, and potential virulence factors were analyzed at the genus level.

## Materials and Methods

### Pangenome and Core Genome Analyses

The genome sequences of thirty-four *Comamonas* strains were retrieved from the IMG database^1^ (Table 1). Among them, the complete genome sequences for *C. kerstersii* 8943, *C. testosteroni* CNB-1 and *C. testosteroni* TK102 are available. Gene clustering was performed by using GET_HOMOLOGUES. The clustering was based on BLASTP and OrthoMCL algorithm (inflation parameter = 1.5). The similar genes required a minimum of 75% coverage with respect to the shortest sequence in the alignment and *E*-value of 1e^-8^ (Udaondo et al., 2017). Due to the unavailability of 16S rRNA gene in some draft genomes, the housekeeping gene *rpoA* was chosen to construct the phylogenetic tree. The *rpoA* gene (about 1 kb) from each genome was aligned by Muscle MEGA using the maximum likelihood tree method with 100 bootstrap replicates (Kumar et al., 2016).

**Table 1:** Genomes of *Comamonas* used in the analysis.

Strain	Origin	Isolation-site characteristics	Genome size (Mb)	GC (%)	Protein coding genes	IMG genome ID	Reference
*C. aquatica* CJG	China	Freshwater river	3.73	64.6	3534	2663763065	Dai et al., 2016
*C. aquatica* DA1877	United States	Soil	3.96	64.8	3464	2576861233	Watson et al., 2014
*C. aquatica* NBRC 14918	–	Fresh water	3.73	65.2	3423	2600255066	Wauters et al., 2003
*C. badia* DSM 17552	Japan	Phenol-adapted activated sludge	3.68	66.0	3489	2528768012	Tago and Yokota, 2004
*C. composti* DSM 21721	China	Food waste compost	4.64	63.2	4075	2524614759	Young et al., 2008
*C. granuli* NBRC 101663	Korea	Granule from bioreactor treating industrial wastewater	3.51	68.5	3191	2600255078	Kim et al., 2008
*C. kerstersii* 8943	China	Clinical samples	3.55	59.6	3272	2772190776	Bioproject: PRJNA378865
*C. kerstersii* J29	China	Isolated from intra-abdominal infection	3.72	59.5	3493	2645727724	Bioproject: PRJNA288932
*C. nitrativorans* DSM 13191	Uruguay	Sludge from denitrifying reactor treating landfill leachate	3.36	63.6	2996	2574180442	Etchebehere et al., 2001
*C. serinivorans* DSM 26136	China	Wheat straw compost	4.52	68.0	3845	2751185825	Zhu et al., 2014
*C. terrae* NBRC 106524	Thailand	Soil contaminated with arsenic	4.71	65.7	4246	2740892221	Bioproject: PRJDB2973
*C. terrigena* NBRC 13299	–	Hay-infusion filtrate	4.63	65.1	4139	2731957557	Wauters et al., 2003
*C. testosteroni* CNB-1	China	Sludge from industrial	5.37	61.0	4803	2561511126	Ma et al., 2009
*C. testosteroni* D4	China	Soil contaminated with arsenic	5.06	61.6	4552	2636415606	Liu et al., 2015
*C. testosteroni* DF1	China	Iron mine soil	5.58	61.1	5173	2636415745	Liu et al., 2015
*C. testosteroni* DF2	China	Iron mine soil	5.57	61.2	5128	2645727942	Liu et al., 2015
*C. testosteroni* DS1	China	Copper-iron mine soil	5.67	61.2	5258	2645727519	Liu et al., 2015
*C. testosteroni* I2	Belgium	Activated sludge	5.78	61.9	5263	2556793030	Boon et al., 2000
*C. testosteroni* JC8	China	Coal mine soil	5.35	61.1	4816	2627853712	Liu et al., 2015
*C. testosteroni* JC9	China	Coal mine soil	5.35	61.1	4797	2648501835	Liu et al., 2015
*C. testosteroni* JC12	China	Coal mine soil	5.36	61.1	4813	2627854154	Liu et al., 2015
*C. testosteroni* JC13	China	Coal mine soil	5.33	61.2	4776	2627854281	Liu et al., 2015
*C. testosteroni* JL14	China	Antimony mine soil	5.74	61.2	5388	2636415849	Liu et al., 2015
*C. testosteroni* JL40	China	Antimony mine soil	5.95	61.1	5492	2630968527	Liu et al., 2015
*C. testosteroni* KF-1	Switzerland	Activated sludge	6.03	61.8	5492	645058871	Weiss et al., 2013
*C. testosteroni* KF712	Japan	Soil contaminated with biphenyl	5.88	61.3	5501	2645727892	Hirose et al., 2015
*C. testosteroni* KY3	Malaysia	Freshwater lake	5.37	61.3	4890	2654587642	Bioproject: PRJNA280733
*C. testosteroni* NBRC 14951	United States	Soil	5.41	61.5	4876	2617271275	Bioproject: PRJDB2973
*C. testosteroni* P19	Korea	Wastewater with biphenyl	5.65	61.5	5124	2687453271	Bioproject: PRJEB10031
*C. testosteroni* TK102	Japan	Soil contaminated with polychlorinated biphenyl	6.06	61.9	5442	2597490204	Fukuda et al., 2014
*C. testosteroni* WDL7	Belgium	Soil	5.50	61.5	4972	2556921102	Dejonghe et al., 2003
*C. testosteroni* ZNC0007	United States	Zebrafish intestine	5.93	61.2	5543	2528311002	Bioproject: PRJNA205594
*C. thiooxydans* DSM 17888	India	Sediment from a sulfur spring	5.27	61.6	4767	2773858010	Pandey et al., 2009
*C. thiooxydans* PHE2-6	Japan	Soil contaminated with trichloroethene	5.30	61.5	4854	2744054452	Shimodaira et al., 2016


### Analysis of Base Composition and Synonymous Codon Usage Bias

To estimate the GC content at the first, second and third nucleotide position of a codon, GC1, GC2 and GC3 were calculated. GC12 represents the average value of the GC content at the first and second positions. ENC value, a measure of codon usage bias for individual gene, was quantified using the SADEG package in R (Khorsand et al., 2017). RSCU value represents the ratio of the observed frequency of the codon relative to the expected frequency under a uniform codon usage for the same amino acid.

### Functional Annotation of Genes in Core- and Pan-Genome

Assignments of genes to COG, KEGG and Pfam database were predicted by WebMGA with COG, KEGG and Pfam databases^2^ (Wu et al., 2011). Virulence related factors were predicted using VFDB^3^, VRprofile^4^, and MP3^5^ (Gupta et al., 2014; Chen et al., 2016; Li et al., 2017). The protein reference database of experimentally-verified virulence factors in VFDB was used. Blastp hits with minimum 40% identity with *E*-value less than 1e^-4^ were considered as positive results. The threshold for the SVM-HMM classifier in the MP3 database was -0.2. Network visualization was conducted using Cytoscape 3.6.1 (Shannon et al., 2003). Membrane transporters were identified using TransportDB 2.0^6^ (Elbourne et al., 2017). Signal transduction genes in the core genome were analyzed based on annotation of *C. testosteroni* CNB-1 in MiST2^7^ (Ulrich and Zhulin, 2009). The predicted transporter and signal transduction genes were verified by the alignment in KEGG and Pfam database.

## Results and Discussion

### Phylogenetic and Comparative Genome Analyses

A total of 34 well-annotated genomes from eleven different *Comamonas* species were chosen to represent the *Comamonas* genus. *C. kerstersii* J29 and 8943 were isolated from clinical samples and all the other strains were isolated from natural environments and anthropogenic processes, including mine soil, activated sludge and compost (Table 1). Intriguingly, the fresh water isolate, *C. aquatica* CJG, also showed potential pathogenic traits such as multiple antibiotic resistance genes as well as the ability to cause sera agglutination (Dai et al., 2016).

**FIGURE 1:**
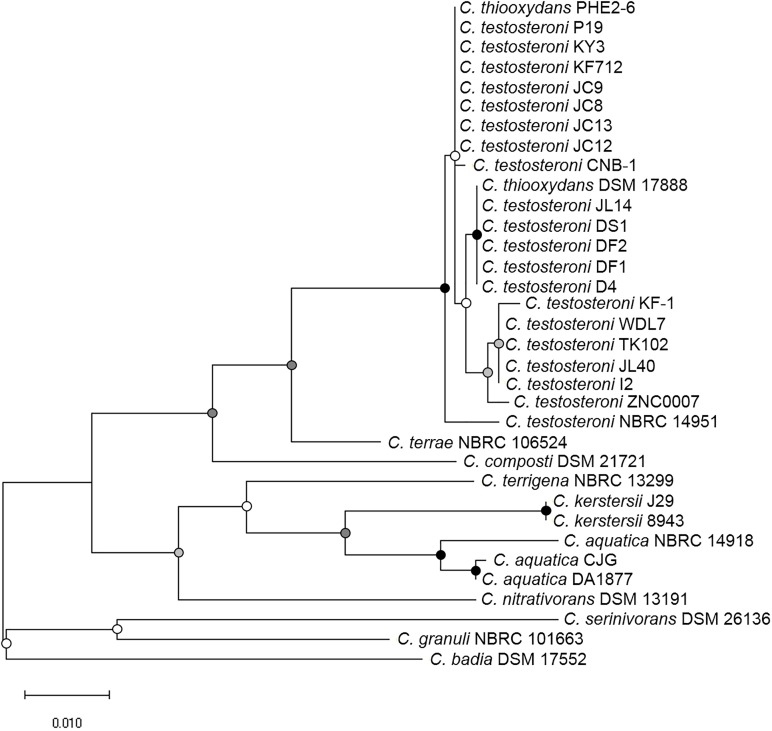
Bootstrapped maximum likelihood phylogenetic tree based on RNA polymerase α subunit (*rpoA*) gene of *Comamonas* species used in this study. The 34 strains were clustered into three subgroups: (1) *C. badia, C. granuli* and *C. serinivorans*; (2) *C. nitrativorans, C. aquatica, C. kerstersii* and *C. terrigena*; (3) *C. composti, C. terrae, C. testosteroni*, and *C. thiooxydans*. The scale indicates the number of substitutions per site. Bootstrap values are indicated by dots at node. Black, >80%; gray, 50–80%; white, <50%.

**FIGURE 2:**
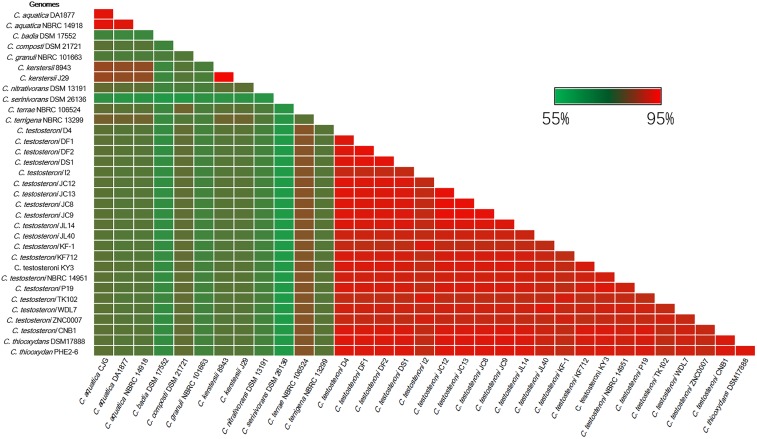
Heatmap indicating percentage of orthologous genes shared by two strains. The identity matrix was calculated based on BLASTP. The average similarity between different species is about 70%. Red gradient bar represents the scale of similarity percentage.

The RNA polymerase α subunit (*rpoA*) gene-based phylogenetic relationship of these 34 strains is shown in Figure 1. The 34 strains separated into three distinct clades. *C. badia* DSM 17552, *C. granuli* NBRC 101663 and *C. serinivorans* DSM 26136 were distinct from the rest. The most sequenced *Comamonas, C. testosteroni* revealed high similarity with *C. thiooxydans*. Meanwhile the clinical isolates, *C. kerstersii* and *C*. *aquatic* were closely related to each other. Further comparative analyses revealed that most of the genomes shared an approximate 70% similarity with other species in the genus (Figure 2). In agreement with Figure 1, *C. serinivorans* DSM 26136 is the most distinct strain, whose genome exhibited a mean similarity of 60.5% with the genomes of the other thirty-three strains. The genomes of the *C. kerstersii* clinical isolates and the potential pathogenic *C. aquatica* strains, exhibited a high similarity of approximately 80%, suggesting that they may share similar virulence-related genomic features. Surprisingly, *C. thiooxydans* strains shared an average similarity of 88.4% with the twenty *C. testosteroni* strains, which is comparable to the similarity between the *C. testosteroni* strains (87.6%). Despite a high similarity in the genome and the 16S rRNA gene sequences, *C. thiooxydans* was defined as a new species because of its thiosulfate oxidation capability (Pandey et al., 2009). Interestingly, all the twenty *C. testosteroni* strains contain sulfur oxidation gene clusters (SOX), suggesting a potential capability of *C. testosteroni* in oxidizing thiosulfate. Hence, considering the high similarity in genomic features and the functional SOX gene clusters, we argue that *C. thiooxydans* may need to be reclassified as *C. testosteroni*.

**FIGURE 3:**
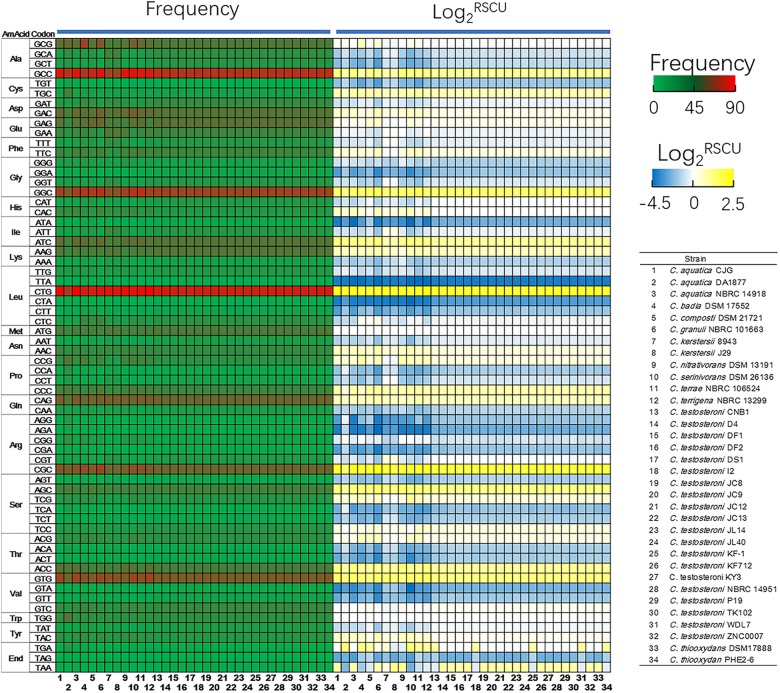
Frequency of codon usage (occurrences per 1000 codons) and relative synonymous codon usage (RSCU) data of *Comamonas* spp. RSCU is the ratio of the observed frequency of the codon to the expected frequency under equal usage of synonymous codons. The codon is more frequently used when Log2-transformed RSCU value higher than 0.

Codon usage is an important determinant in evolution. The base composition of protein-coding genes revealed that only *C. kerstersii* J29 has a GC content below 60% and *C. granuli* NBRC 101663 has the highest GC content of 68.44 ± 4.47% (Supplementary Table S2). The reduced GC content for the clinical isolate could be attributed to the conservation of replication expense in nutrient-limiting environments (Rocha and Danchin, 2002). With the lowest effective number of codons (ENC) of 38.86 ± 4.59, genes of *C. granuli* NBRC 101663 exhibited a strong bias in codon usage pattern. The ENC values for other environmental strains ranged from 40.20 to 49.20, while the average value for *C. kerstersii* strains was up to 51.46, suggesting more low-codon-biased genes in these strains. Based on the relative synonymous codon usage (RSCU) (Figure 3), *Comamonas* spp. prefer certain codons. GGC (Gly), CTG (Leu), CGC (Arq), AGC (Ser), and GTG (Val) were more frequently used, which had RSCU values higher than 2 across all the 34 *Comamonas* strains.

### Pangenome Analysis of *Comamonas*

All the protein-coding genes in the 34 genomes were clustered into 22,599 gene clusters (i.e., the pangenome). Among them, 1,009 gene clusters composed the core genome. Different genes were clustered when the encoded protein sequence coverage was higher than 75% coverage, with a cut-off less than 1E^-8^ (Udaondo et al., 2017). At different similarity thresholds, the core genome sizes were consisted of 1,190 gene clusters for 25% coverage and 1,146 for 50% coverage (Supplementary Figure S1A). Reduction of similarity thresholds from 75 to 25% resulted in an increase of the core gene clusters percentage in the pangenome from 4.46 to 6.74%. In the pangenome, 7.54% of gene clusters were conserved in more than 32 genomes (>94% of all taxa), 26.24% of gene clusters were shared by 3–31 genomes, and 66.69% were only present in 1 or 2 genomes (<6% of all taxa) (Supplementary Figure S1B). Collectively, these results suggest the presence of a significant amount of conserved and highly specific genes in all the 34 *Comamonas* strains.

**FIGURE 4:**
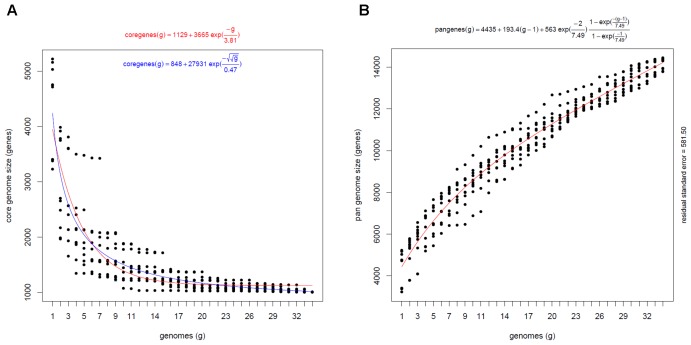
Statistic estimation of the core genome **(A)** and pangenome **(B)** size of *Comamonas* genus. Core genome size was estimated with Tellelin (Red) and Willenbrock (Blue) fit. The number of core gene clusters approximated to 1000. Pangenome size was estimated with Tettelin fit. 193 gene clusters were added to the pool with per genome inclusion. The continuous curve represents the least-squares fit of the corresponding exponential functions.

Based on the statistical estimation, the analysis of the increasing number of genomes converged the core genome size to about 1000 gene clusters (Figure 4A). In contrast, an average of approximately 193 strain-specific gene clusters were added into the pangenome with the inclusion of each additional genome (Figure 4B). It indicates that every *Comamonas* strain encodes a certain number of unique genes and the *Comamonas* genus possesses an open pangenome, meaning that the analyzed strains contain certain unique genes which are not shared by other strains and the gene pool size would continue to increase with the increasing number of genomes incorporated in the analysis. This pan-genomic feature enables different strains to survive in diverse environmental niches.

### Central Carbon Metabolism of *Comamonas*

**FIGURE 5:**
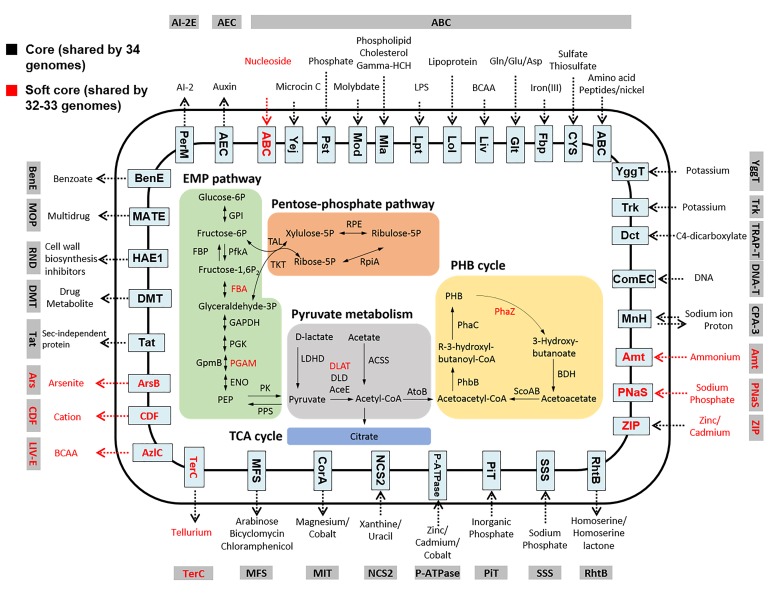
Overview of central carbon metabolism and transporters in *Comamonas*. Transporters are grouped into different families. Gene products shared by all the 34 genomes (core genome) and 32–33 genomes (soft core genome) are shown in black and red, respectively. *Comamonas* possess conserved pathways to metabolize phosphorylated glucose and to store the assimilated carbon as PHB. EMP pathway: Embden-Meyerhof-Parnas pathway. Genes are referred in commonly used names. GPI, glucose-6-phosphate isomerase; FBA, fructose-bisphosphate aldolase; GAPDH, glyceraldehyde 3-phosphate dehydrogenase; PGK, phosphoglycerate kinase; PGAM, 2,3-bisphosphoglycerate-dependent phosphoglycerate mutase; ENO, enolase; PK pyruvate kinase. PEP, Phosphoenolpyruvate.

The analyses of the core genome of the *Comamonas* genus revealed conserved acetate and pyruvate metabolic pathways across different *Comamonas* species (Figure 5). In particular, the presence of the poly-β-hydroxybutyrate (PHB) cycle in the central carbon metabolism pathway suggests that *Comamonas* has the capability of accumulating carbon in the form of PHB granules. Based on BLAST analysis (Supplementary Figure S2), these enzymes were assigned as class I PHA synthases which preferentially utilize short-carbon-chain-length hydroxyalkanoic acid CoA thioesters as substrate (Bernd, 2003). Although many *Comamonas* strains have been reported to be able to degrade aromatic compounds, no core genes were assigned to the xenobiotic biodegradation function by KEGG annotation. This suggests that the catabolic pathways are not well conserved at the genus level. Our previous studies have demonstrated that a number of catabolic genes are present in the plasmids (Wu et al., 2006, 2015a).

Although the core genome lacks key genes for glucose phosphorylation and the phosphotransferase system, putative enzymes such as glucose-6-phosphate isomerase (GPI), 6-phosphofructokinase 1 (PfkA) and fructose-bisphosphate aldolase (FBA) encoded in the core genome are capable of metabolizing phosphorylated glucose (e.g., glucose-6P). In addition, 23 of the 34 *Comamonas* genomes also contain the gene encoding sugar phosphate permease. These transporters may enable *Comamonas* to uptake phosphorylated sugar from the environment (Chico-Calero et al., 2002). Unlike *P. putida* and many other environmental bacteria, the core genome of *Comamonas* does not code putative enzymes for the Entner Doudoroff pathway for glucose metabolism. Similar core genomic features were observed based on the COG functional classification (Supplementary Figure S3). Approximately 140 proteins from each genome were predicted for amino acid metabolism, in comparison to 31 proteins for carbohydrate metabolism. Consistent with the core genome analyses, *Comamonas* strains have been reported to grow well on various amino acids and organic acids, but rarely catabolize pentoses or hexoses (Willems and Vos, 2006). Consequently, when the same inoculum was introduced to microbial fuel cells, *Comamonas* was enriched in the acetate-fed but not in the glucose-fed system (Xing et al., 2009).

### Role in Nitrate Reduction

**FIGURE 6:**
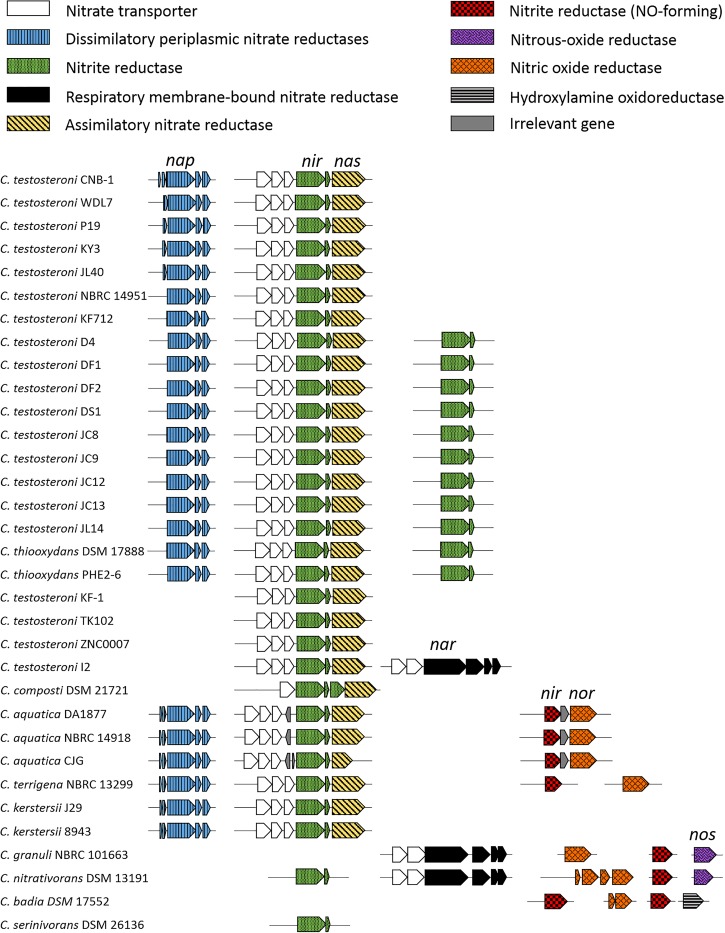
Comparison of nitrogen metabolism in *Comamonas* spp. Most strains are capable of nitrate reduction via ammonification or denitrification. *nap*, periplasmic nitrate reductase; *nir*, nitrite reductase; *nas*, assimilatory nitrate reductase; *nar*, nitrate reductase; *nirA*, ferredoxin-nitrite reductase; *nor*, nitric oxide reductase.

In previous studies, *Comamonas* has often been reported to be enriched in nitrate-reducing conditions (Patureau et al., 2000; Guo et al., 2015; Zhong et al., 2015), suggesting a potential role of *Comamonas* in nitrate reduction. Genomic analyses revealed highly diverse nitrogen metabolism pathways in *Comamonas* (Figure 6). *C. terrae* NBRC 106524 is the only strain which does not contain any genes involved in nitrate or nitrite metabolism. The genomes of *C. nitrativorans* DSM 13191 and *C. granuli* NBRC 101663 contain all the genes required for complete denitrification. *C. aquatica* and *C. terrigena* strains are capable of reducing nitrate to nitrous oxide. Except *C. terrae, C. badia*, and *C. serinivorans*, each of the rest thirty-one genomes harbors at least one copy of the nitrate reductase gene. Twenty-nine genomes including those of *C. testosteroni, C. thiooxydans, C. composti, C. aquatica, C. terrigena*, and *C. kerstersii* contain an analogous gene cluster for ammonification which comprises one assimilatory nitrate reductase gene (*nas*) and at least two nitrite reductase genes (*nir*). Twenty-five among them also contain one dissimilatory nitrate reductase gene. Therefore, *Comamonas* spp. are capable of assimilatory and dissimilatory reduction of nitrate. The resultant nitrite can be further reduced to ammonia by nitrite reductase (Nir).

Due to the high abundance in denitrifying microbial communities and denitrification capability of specific isolates, *Comamonas* spp. were considered to be potential denitrifiers (Lemmer et al., 1997; Etchebehere et al., 2001; Gumaelius et al., 2001). Our results confirmed the genomic potential of the *Comamonas* genus in nitrate reduction. Previous studies found that the increased ratio of electron donors to electron acceptors can stimulate the ammonification route relative to denitrification (King and Nedwell, 1987). When the competition for nitrate becomes more severe, the decreased nitrate levels would favor ammonifying bacteria over denitrifying bacteria (Dong et al., 2009). Moreover, the ATP synthesis in denitrification has been shown to be lower than in ammonification (Strohm et al., 2007). Therefore, *Comamonas* strains may gain a competitive advantage from ammonification over other denitrifiers in nitrate-reducing conditions. Generally, *Comamonas* are considered aerobes, although the strains with the Nar-type nitrate reductases are facultative anaerobes capable of respiring under anoxic conditions (Wu et al., 2015b).

### Sophisticated Environmental Sensing and Signaling Systems

The core genome analysis of the *Comamonas* genus revealed 39 gene clusters with potential roles in microbial signal transduction, including 25 for the one-component systems, 12 for the two-component systems, and 2 for extracytoplasmic function (ECF). Per-Arnt-Sim (PAS) is the most abundant sensor domain in two-component systems while LysR substrate-binding domain is the most abundant in one-component systems. With PAS domains, *Comamonas* strains are capable of mediating the responses to changes in redox state and cellular oxygen level (Krell et al., 2010). Other than *C. granuli* and *C. nitrativorans*, other *Comamonas* strains harbor putative genes for three types of oxygen-dependent terminal oxidases, among which the *cbb3*-type and *bd*-type are abundant. As the *cbb3*-type and *bd*-type oxidases have a high oxygen affinity (Alvarez-Ortega and Harwood, 2007), this combination facilitates growth of *Comamonas* under conditions with varying oxygen levels. This could be one reason why *Comamonas* was often found to be highly abundant in bioprocesses with intermittent aeration (e.g., EBPR reactors) (Liu et al., 2005; Zhang et al., 2005; Zengin et al., 2011).

### Signaling System Involved in Biofilm Development

The GGDEF domain proteins are essential for c-di-GMP synthesis, while EAL and HD-GYP domain proteins break down this signaling molecule (Hengge, 2009). Based on Pfam annotation, there is no c-di-GMP controlling gene conserved across the 34 genomes. Except for *C. serinivorans*, many genes (21–60) which control c-di-GMP are present (Supplementary Figure S4). The percentage of c-di-GMP controlling genes range from 0.5 to 1.7% of total protein-coding genes. *C. composti* which shared a similar environmental source with *C. serinivorans* encoded the lowest number of these catalytic proteins. Meanwhile, the ratio of c-di-GMP controlling genes in all the other thirty-two genomes are above 0.75%, which is higher than 0.6% in other environmental bacteria such as *E. coli* K12 and *P. putida* KT2440 (Wu et al., 2015a). Among these putative c-di-GMP controlling proteins, about 45% of them have binding capacity to a sensory domain that enables effective c-di-GMP level regulation. Although many genes involved in c-di-GMP signaling were identified among the thirty-three strains, only one conserved gene encoding HD-GYP domain-containing protein was found. It suggests that the c-di-GMP controlling genes are diversified across different species in *Comamonas* genus.

Quorum sensing (QS) is another central signaling system for biofilm regulation in which bacterial cells communicate through secreted signaling molecules. Although no QS signal synthase genes were found in the core genome, two genes coding for anthranilate synthase (*phnA* and *phnB*) were identified. Anthranilate synthase has been reported to be exclusively for synthesizing the precursor of QS signals in *Pseudomonas* (Farrow and Pesci, 2007). The presence of anthranilate synthase genes in the core genome of *Comamonas* suggests that *Comamonas* may produce QS signals through similar pathways.

### Virulence Factors in *Comamonas*

**FIGURE 7:**
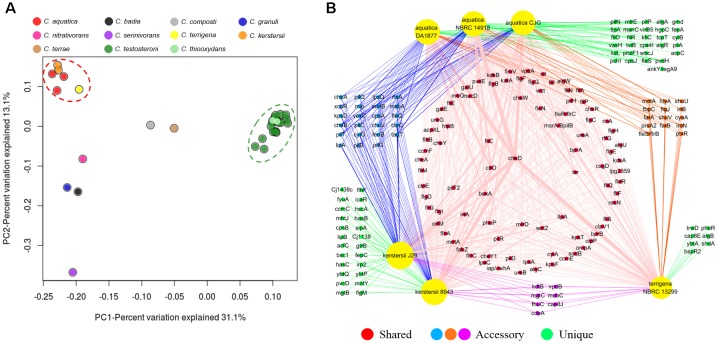
PCoA plot illustrates the differences between virulence gene orthologs in *Comamonas* spp. **(A)**. The network analysis reveals the shared (present in three species), accessory (present in two species) and unique (present in one species) virulence factors in *C. terrigena, C. aquatica*, and *C. kerstersii* **(B)**. The width of each edge is proportional to the number factors in the genome.

Since 1987, *Comamonas* had been found to be associated with certain human infections (Barbaro et al., 1987). To analyze the pathogenic potential of *Comamonas*, virulence factors in the genomes were identified by BLAST search against the Virulence Factors Database (VFDB)^8^. On average, each genome of *Comamonas* contains about 200 virulence factors while no conserved virulence factor was found in the core genome. Principal coordinates analysis (PCoA) of virulence factors in each genome revealed two distinct clusters (Figure 7A). Similar clustering patterns were also observed for the virulence factors predicted in MP3 and VRprofile (Supplementary Figure S5). One consists of the clinically isolated *C. kerstersii*, potentially pathogenic *C. aquatica* and environmental *C. terrigena*, while the other consists of all the *C. testosteroni* and *C. thiooxydans* strains. These species, especially *C. kerstersii* and *C. testosteroni*, are most frequently isolated from clinical samples (Opota et al., 2014). *C. terrigena* was also reported to be associated with bacteraemia which reveals a close phylogenetic relationship with *C. kerstersii* and *C. aquatica* (Figure 1; Sonnenwirth, 1970). Although the genomic information of clinically isolated *C. testosteroni* is not available, the close similarity between strains from various origins indicates the species share certain virulence factors. To explore the conserved potential virulence mechanisms for *Comamonas*, the virulence factors of strains in the two clusters which were identified by VFDB were further analyzed.

There are 87 virulence factors shared by *C. kerstersii, C. aquatica*, and *C. terrigena*. 44 factors are conserved by two of them and 60 factors are unique which were only predicted in one species (Figure 7B). The shared offensive virulence factors comprise a number of genes responsible for bacterial motility and adherence. The flagellar and pilus assembly pathways and chemotaxis signaling system are well-conserved in these three species, in which multiple genes encoding methyl-accepting chemotaxis proteins (Tsr) were identified (Supplementary Table S3). The shared biosynthesis genes of capsular polysaccharide, lipopolysaccharide, alginate and adhesins, like IlpA, Hsp60, P60, and P5, suggest the adherence and biofilm formation capability of these species. Although no shared toxin protein was predicted, *hlyA* or *rtxA* which encode hemolysin are present in five of these six strains (83.3%). The shared defensive and nonspecific (i.e., neither offensive nor defensive) virulence factors consist of various stress proteins and metabolic enzymes. ClpEP (phagosome), SodB (SOD), RecN (ROS, neutrophils), Urease (acidity), and MsrAB (ROS) are able to protect cellular functions against stress. The ability to source for nutrients is key for survival and multiplication in the host. The putative isocitrate lyase (Icl) enables them to use fatty acids as the carbon and energy source.

For the other cluster composed of *C. testosteroni* and *C. thiooxydans*, 108 factors are shared by these 22 genomes (Supplementary Figure S6). Fifty-seven factors are unique and shared by only one or two strains (Supplementary Table S4). Similar to the previous cluster, *C. testosteroni* and *C. thiooxydans* also encode a number of genes for motility, polysaccharide and adhesin biosynthesis. The pilus assembly and polysaccharide biosynthesis genes are more abundant in this cluster. Each genome also contains an average of 5 *bspR2* genes which encode putative quorum-sensing regulatory proteins. It indicates that *C. testosteroni* and *C. thiooxydans* strains may have better biofilm-forming capabilities. Meanwhile, these species may be less cytotoxic than *C. kerstersii* and *C. aquatica*, as only 14 out of 22 genomes (63.6%) contain the hemolysin gene. With respect to the defensive and nonspecific virulence factors, more genes shared in this cluster encode putative metal uptake proteins, especially for various siderophores.

In summary, the virulence factors of the *Comamonas* genus are very diverse. Meanwhile, clinically isolated and potentially pathogenic strains shared specific virulence factors, including polysaccharide biosynthesis for adherence and anti-phagocytosis, motility system and metabolic enzymes for adaptation *in vivo*. Although no related pathogenic effect on healthy people caused by *Comamonas* strains has been reported, all the clinically isolated *Comamonas* strains and a number of environmental *Comamonas* contain hemolysin genes. Therefore, the core- and pan-genomic analyses revealed that potential virulence may be species-specific as certain virulence factors are conserved in pathogenic-like strains.

## Conclusion

*Comamonas* is a group of ubiquitous bacteria present in various natural and engineered environments. Some of them are also involved in a number of clinical cases. It has been suggested that *Comamonas* strains may share specific genomic features at the genus level and play certain ecological roles to different habitats. The pan-genomic analysis shows the diverse genomic features that contribute to the wide adaptation of the genus to various environments. The core genome reveals central metabolic pathways that enable *Comamonas* to utilize various nutrient sources and store excess resources as PHB. The conserved dissimilatory and assimilatory nitrate reductases in *Comamonas* explain their presence in nitrate reducing environments and suggest an important role in the nitrogen biogeochemical cycle. They also encode sophisticated redox sensory systems and effective c-di-GMP controlling systems, allowing them to adjust their biofilm lifestyle under dynamic conditions. The virulence factors in *Comamonas* are found to be highly species-specific. The conserved mechanisms for potentially pathogenic *Comamonas* are related to surface adherence, motility control, nutrient acquisition and stress tolerance.

## Author Contributions

YW and BC conceived this study and wrote the manuscript. YW and NZ performed the analyses. All authors read and approved the final manuscript.

## Conflict of Interest Statement

The authors declare that the research was conducted in the absence of any commercial or financial relationships that could be construed as a potential conflict of interest.

## Footnotes

1 https://img.jgi.doe.gov/

2 http://weizhong-lab.ucsd.edu/metagenomic-analysis/

3 http://www.mgc.ac.cn/VFs/

4 http://bioinfo-mml.sjtu.edu.cn/VRprofile/

5 http://metagenomics.iiserb.ac.in/mp3/

6 http://www.membranetransport.org/

7 http://mistdb.com

8 http://www.mgc.ac.cn/VFs/
