# Supplementary material for: The Core- and Pan-Genomic Analyses of the Genus Comamonas: From Environmental Adaptation to Potential Virulence
Source: Front Microbiol. 2018 Dec 12;9:3096. doi: 10.3389/fmicb.2018.03096 (PMC6299040; doi:10.3389/fmicb.2018.03096)
Supplement: Supplementary file 1 [file Data_Sheet_1.PDF]

Supplementary Material

The core- and pan-genomic analyses of the Genus *Comamonas*: From environmental adaptation to potential virulence

Yichao Wu [1], Norazean Zaiden [2], Bin Cao [*,2,3]

[1]State Key Laboratory of Agricultural Microbiology, College of Resources and Environment, Huazhong Agricultural University, Wuhan, China

[2]Singapore Centre for Environmental Life Sciences Engineering, Nanyang Technological University, Singapore

[3]School of Civil and Environmental Engineering, Nanyang Technological University, Singapore

*Correspondence:

Bin Cao

bincao@ntu.edu.sg

Table S1. The abundance of *Comamonas* in different environments without aromatic pollutants.

Reactor type	Samples	*Comamonas* abundance	Quantification method	Comments	Reference
Sewage treatment plants	Activated sludge	1.0 %	16s rDNA amplification		(Zhang, Shao et al. 2012)
Sequencing batch reactor	Phosphate-removing activated sludge	3.5 %	16s rDNA amplification		(Liu, Zhang et al. 2005)
Sequencing batch reactor	Phosphate-removing activated sludge	5.8 %	16s rDNA amplification		(Zhang, Liu et al. 2005)
Sequencing batch reactor	Activated sludge	43.8 %	16s rDNA amplification	Without accumulation of polyphosphate and glycogen	(Fang, Zhang et al. 2002)
Sequencing batch reactor	Phosphate-removing Activated sludge	1.0 %	16s rDNA amplification		(Zengin, Artan et al. 2011)
Granular sludge reactor	Sulfate-reducing sludge	5.8 %	16s rDNA amplification	Detected in the substratum zone	(Hao, Luo et al. 2016)
Anaerobic rising main pipes	Biofilm on plastic carriers	6.4%	16s rDNA amplification	Increased after nitrate dosage	(Auguet, Pijuan et al. 2015)
Biological aerated filter reactors	Biofilm community on substratum	3.6-40.1 %	16s rDNA amplification		(Kim, Pagaling et al. 2014)
Water lifting and aeration system	Water sample	29.4 %	16s rDNA amplification	Nitrogen removing system	(Zhou, Huang et al. 2016)

Activated sludge system	Activated sludge	4.4-4.3 %	16s rDNA amplification	Low ammonium and oxygen condition	(Sui, Liu et al. 2016)
Single-chamber microbial fuel cells	Anode biofilm	>5 %	16s rDNA amplification		(Jiang, Xing et al. 2016)
Cr(VI)-contaminated Soil	Surface soil	4 %	16s rDNA amplification		(Wang, Peng et al. 2015)
Wetland	Typha rhizosphere community	8.8 %	16s rDNA amplification		(Guo, Gong et al. 2015)
Microbial fuel cell cascade	Anodic biofilm	5.4 %	16s rDNA amplification		(Hodgson, Smith et al. 2016)
Microbial fuel cell	Anodic biofilm	7.0 %	16s rDNA amplification		(Borole, Hamilton et al. 2009)
Microbial fuel cell	Cathodic microbial community	13.2 %	16s rDNA amplification	Intermittent aerated cathode	(Sotres, Cerrillo et al. 2016)
Biofilm development on glass slide	Biofilm	>10 %	16s rDNA amplification	Slime on river stones as inoculum	(Nakamura, Yamamoto et al. 2016)

Table S2. Base composition and the effective number of codons (ENC) of protein-coding genes in *Comamonas* strains

	GC (%)	GC1 (%)	GC2 (%)	GC12 (%)	GC3 (%)	ENC
C. aquatica CJG	64.89+5.48	70.18+6.68	45.76+5.71	57.97+5.12	82.94+10.37	43.00+6.38
C. aquatica DA1877	65.43+5.31	70.33+6.42	45.74+5.22	58.03+4.88	83.40+10.28	42.65+6.23
C. aquatica NBRC 14918	65.33+5.01	70.36+6.36	45.88+5.43	58.12+4.84	83.92+9.23	42.39+5.71
C. badia DSM17552	66.14+4.01	71.26+5.86	47.10+5.25	59.18+4.64	83.24+7.05	43.05+4.84
C. composti DSM21721	63.70+4.78	69.37+6.31	46.16+5.21	57.76+4.76	78.59+8.72	46.11+5.19
C. granuli NBRC101663	68.44+4.47	72.92+6.49	47.44+5.58	60.18+5.25	89.26+6.78	38.86+4.59
C. kerstersii 8943	60.17+4.45	68.15+6.19	44.44+5.09	56.29+4.50	70.69+8.52	51.44+4.37
C. kerstersii J29	59.72+4.77	67.96+6.46	44.40+5.17	56.18+4.63	70.42+9.04	51.48+4.47
C. nitrativoran DSM13191	64.14+5.33	69.85+6.42	45.62+5.20	57.74+4.87	80.74+9.89	44.47+5.74
C. serinivorans DSM 26136	67.99+4.41	71.78+6.18	48.15+6.18	59.96+5.10	87.32+7.16	40.20+4.94
C. terrae NBRC 106524	66.19+4.67	70.82+6.27	46.90+5.46	58.86+4.84	84.24+8.79	42.19+5.47
C. terrigena NBRC 13299	65.37+4.61	70.79+6.49	46.64+5.64	58.71+5.01	81.86+7.76	43.86+4.64
C. testosteroni D4	61.87+4.11	68.44+5.62	45.48+5.05	56.96+4.17	75.44+8.05	48.43+4.94
C. testosteroni DF1	61.25+4.76	67.87+6.08	45.43+5.21	56.65+4.48	74.13+9.25	49.11+5.28
C. testosteroni DF2	61.28+4.76	67.91+6.04	45.42+5.21	56.67+4.47	74.19+9.25	49.07+5.27
C. testosteroni DS1	61.28+4.81	67.83+6.09	45.52+5.25	56.68+4.48	74.20+9.32	49.05+5.30
C. testosteroni I2	62.28+4.66	68.30+6.01	45.72+5.24	57.01+4.42	75.73+9.24	48.02+5.35

C. testosteroni JC8	61.43+4.53	68.09+5.84	45.43+5.09	56.76+4.30	74.44+9.05	48.95+5.26
C. testosteroni JC9	61.44+4.53	68.14+5.79	45.42+5.08	56.78+4.30	74.44+9.05	48.95+5.25
C. testosteroni JC12	61.43+4.52	68.11+5.80	45.42+5.07	56.77+4.29	74.43+9.04	48.96+5.25
C. testosteroni JC13	61.47+4.51	68.12+5.85	45.44+5.10	56.78+4.30	74.52+8.99	48.90+5.22
C. testosteroni JL14	61.22+4.70	67.87+6.00	45.47+5.19	56.67+4.38	74.05+9.28	49.20+5.43
C. testosteroni JL40	61.13+5.02	67.72+6.17	45.43+5.27	56.58+4.52	73.92+9.90	49.15+5.59
C. testosteroni KF712	61.39+4.71	68.03+5.89	45.49+5.23	56.76+4.42	74.36+9.33	48.95+5.32
C. testosteroni KF-1	62.20+4.72	68.26+6.00	45.70+5.13	56.98+4.39	75.53+9.38	48.16+5.48
C. testosteroni KY3	61.47+4.57	68.11+5.87	45.37+5.12	56.74+4.35	74.61+9.00	48.82+5.16
C. testosteroni NBRC 14951	61.73+4.28	68.28+5.85	45.60+5.14	56.94+4.27	75.01+8.50	48.67+5.12
C. testosteroni P19	61.64+4.46	68.24+5.79	45.57+5.12	56.91+4.30	74.79+8.83	48.75+5.19
C. testosteroni TK102	62.16+4.65	68.49+5.90	45.86+5.22	57.18+4.41	75.85+9.11	47.98+5.40
C. testosteroni WDL7	61.97+4.45	68.14+5.88	45.60+5.24	56.87+4.33	75.05+8.93	48.57+5.29
C. testosteroni ZNC0007	61.23+5.54	67.73+6.44	45.44+5.43	56.58+4.82	74.24+10.68	48.72+5.65
C. testosteroni CNB1	62.02+4.31	68.39+5.69	45.46+5.15	56.93+4.25	75.01+8.78	48.59+5.09
C. thiooxydans DSM17888	61.84+4.2	68.34+5.66	45.55+5.04	56.95+4.20	75.33+8.31	48.47+5.03
C. thiooxydans PHE2-6	61.94+4.22	68.24+5.68	45.46+5.08	56.85+4.20	75.02+8.39	48.65+4.97

Table S3. Virulence factors in *C. kerstersii*, *C. aquatica* and *C. terrigena* strains.

Category	Virulence Factor	Shared	Accessory	Unique
Capsule synthesis	Capsule I (polysaccharide)	*wzt2* (16)	*vexC* (5),	*tviB* (1), *manC* (2)
	Capsule	*kpsFT* (11), *ctrD* (37), *bexA* (28)	*cap8J* (3), *cpsA* (4), *kpsD* (4), *lipA* (5)	*bsc1* (2), *cap8E* (1), Cj1138 (4), Cj1438c (1), *cpsB4IJ* (6), *hscA* (1), *lipB* (1)
	Hyaluronic acid capsule			*hasBC* (2)
Adhesin and surface characteristics	IlpA	*ilpA* (12)		
	Hsp60	*htpB* (8)		
	ICA			*icaR* (2)
	PI-2			*sipA* (2)
	Mip		*mip* (5)	
	PrsA2		*prsA2* (3)	
	P60	*iap/cwhA* (6)		
	LPS	*acpXL* (6), *fabZ* (6), *rffG* (7), *orfM* (6), *lpxAC* (13), *galEU* (10), *kdsAB* (12), *wzt* (7)	*kdtB* (3), *lpxD* (3), *msbA* (5)	*kfiC* (1), *gtrB* (2), *acpC* (1), *wbcJ* (1), *gmd* (1)
	fimbriae	*csgD* (9), fimV (6)		
	ShdA			*shdA* (1)
	P5 protein	*ompA* (6)		
	ACF			*acfC* (2)
	Alginate	*algCUW* (19), *mucD* (7)		*algABR* (4)
Motility	flagella	*fleNR* (27), *motA* (6), *cheADRWYY1* (134), *fliACEFGIMNPQS* (88), *tsr*	*cheB* (8), *fleQS* (10), *flgJ* (5), *motB* (5)	*fleS* (1), *flgM* (2), *flhF* (2), *fliOR* (4), *motY* (2), *tlpA* (3)

		(18), *flgBCDEFGHI* (53), *flhABC* (18)		
	Type IV pili	*pilDHRT2* (36)	*chpA* (5), *pilBCGQT* (25)	*pilANP* (6), *tcpT* (1)
Signaling	c-di-GMP controlling		*cdpA* (3)	
	Two-component system	*phoP* (15)		*phoR* (1)
	Quorum-sensing			*bspR2* (1)
Metal uptake	Yersiniabactin			*ybtAPQ* (5), *irp2* (4), *fyuA* (2)
	Pyoverdine	*pvdE* (6)	*ptxR* (4)	*pvdH* (5), *pvcD* (2)
	Pyochelin			*pchI* (1)
	Mycobactin	*irtA* (11)	*irtB* (3)	*mbtJ* (2)
	iroN		*iroN* (6)	
	Enterobactin			*fepAC* (2)
	FbpABC		*fbpC* (2)	
	Shu-heme		*shuUV* (5)	
	ccmc	*ccmEF* (12)		*ccmC* (2)
	Chu		*chuV* (5)	
	FeoAB		*feoB* (5)	
	MobABC		*mobC* (3)	
	MgtBC		*mgtC* (3)	*mgtB* (2)
	MntABC		*mntA* (5)	
	FhuCBD		*fhuC* (3)	
	hemoglobin-binding protein			*hgpC* (1)

Metabolism	urease	*ureABG* (23)		
	Isocitrate lyase	*icl* (6)		
Efflux pump	MtrCDE	*mtrCD* (24)		*mtrE* (4)
	FarAB		*farAB* (9)	
Stress protein	RecN	*recN* (6)		
	SodB	*sodB* (6)		
	MsrAB	*msrA/B*(*pilB*) (15)		
	Clp	*clpEP* (12)	*clpC* (3)	
	KatAB	*katA* (7)		
Secretion and transport system	Bsa T3SS Type III secretion	*bprAB* (21)		
	Type IV secretion	*lpg2359* (6), *vpdA* (4)	*vpdB* (3)	*ankI/legAS4* (1), *trwD* (1), *lidL* (2), *virB5* (1)
	Type VI secretion	*clpV1*(6)	*tagT* (11)	*vasH* (3)
Toxin related	RTX toxin		*rtxA* (4)	
	Hemolysin		*hlyA* (3)	
	Cytolysin	*cylA* (6)	*cylG* (8)	*cylB* (1)
	CYA		*cyaA* (2)	
	xcp Type II secretion system		*xcpR* (5)	
Other virulence factor	RicA	*ricA* (6)		
	Phenazines			*phzF1* (2)

Table S4. Virulence factors in *C. testosteroni* and *C. thiooxydans* strains.

Category	Virulence Factor	Shared	Accessory	Unique
Capsule synthesis	Capsule I (polysaccharide)	*wzt2* (102), *vexC* (23)	*manC* (7), *tviB* (9)	*wcbABCDOT* (6), *tviC* (1)
	Capsule	*kpsFT* (64), *ctrD* (74), *bexA* (38), *cpsA* (22)	*cap8E8F8J* (15), *cps4E4IBJ* (65), *kpsD* (15), *lipA* (15), *wlaN* (3)	*bsc1* (1), *cap8P* (7), Cj1138 (1), Cj1438c (2), Cj1440c (1), *cpsO* (1)
	Hyaluronic acid capsule		*hasBC* (13)	
Adhesin and surface characteristics	IlpA	*ilpA* (30)		
	Hsp60	*htpB* (22)		
	PrsA2	*prsA2* (22)		
	LPS	*acpXL* (22), *fabZ* (22), *rffG* (23), *orfM* (22), *lpxC* (22), *galEU* (44), *kdsAB* (54), *wzt* (34), *kdtB* (22)	*bplL* (9), *lpxA* (29), *gtrB* (10), *msbA* (18), *bplBC* (15), *gmd* (3)	*wbcJ* (2), *wzm* (1), *gmhA* (1), *pmm* (1), *lpxE* (1), *ddhAB* (2)
	fimbriae	*fimV* (22)	*csgD* (23), *daaC* (16), *papD* (3)	*fimBE* (2)
	ShdA		*shdA* (6)	
	P5 protein	*ompA* (22)		
	Lpf (Long polar fimbriae)		*f17d-D* (3), *lpfA* (3)	
	ACF		*acfC* (21)	
	Alginate	*algBCRUWZ* (177), *mucD* (44)		
Motility	flagella	*fleNQ* (76), *motAB* (44), *cheABDRWYY1* (413), *fliACEGIMNPRS* (261), *tsr*	*fleRS* (48), *flgDH*(37), *fliFO* (29), *tlpA* (19), *cheZ* (5)	*fliQ* (1), *cheV3* (1), *pseI* (1)

		(70), *flgBCEFGIJR* (184), *flhABC* (66)		
	IcsA (VirG)		*icsA/virG* (5)	
	Type IV pili	*pilBCDGHMTT2* (199), *chpAE* (45)	*pilAEQRX* (77)	*pilLUVZ* (8)
Signaling	c-di-GMP controlling			*cdpA* (2)
	Two-component system	*phoP* (62)		
	Quorum-sensing	*bspR2* (112)	*bspR1* (5)	
Metal uptake	Yersiniabactin		*ybtQ* (4), *irp2* (3)	*ybtAP* (2)
	Pyoverdine		*ptxR* (3), *fpvA* (22)	*pvdI* (2)
	Pyochelin	*pchD* (22)	*pchE* (5), *fptA* (3)	*pchF* (1)
	Mycobactin	*irtA* (44)	*mbtC* (21),	*mbtBEJ* (4), *irtB* (2)
	iroN	*iroN* (22)		
	Enterobactin		*fepBCG* (46)	*entE* (2), *fepA* (2)
	FbpABC	*fbpC* (31)		
	Shu-heme		*shuSV* (22)	*shuU* (1)
	ccmc		*ccmBEF* (58)	
	Chu	*chuV* (22)		*chuT* (1)
	FeoAB		*feoB* (17)	
	MobABC	*mobC* (22)		
	MgtBC	*mgtB* (26)	*mgtC* (4)	
	MntABC		*mntA* (24)	
	FhuCBD		*fhuC* (14)	

	HxuABC		*hxuC* (3)	
Metabolism	urease	*ureABG* (88)		
	Isocitrate lyase	*icl* (24)		
Efflux pump	MtrCDE	*mtrCD* (90)		
	FarAB	*farAB* (51)		
Stress protein	RecN		*recN* (14)	
	SodB	*sodB* (27)		
	MsrAB	*msrA/B*(*pilB*) (90)		
	Clp	*clpEP* (50)		*clpBC* (2)
	CAMP			*eptC* (1)
	CβG			*cgs* (1)
	KatAB	*katA* (22)		
Secretion and transport system	Type II secretion		*lspK* (5)	*gspE* (1)
	Bsa T3SS Type III secretion	*bprAB* (109)		*pscC* (1)
	Type IV secretion	*lpg2359* (22), *vpdB* (22), *lidL* (82)		*virB4/cagE* (1), *virB9/cagX* (1), *lpnE* (1)
	Type VI secretion	*tagT* (29), *clpV1* (23)	*vasH* (19)	*vgrG-2* (1)
Toxin related	RTX toxin		*rtxA* (14)	*rtxB* (1)
	Hemolysin			*hlyB* (2)
	Cytolysin	*cylAB* (45)	*cylG* (21)	
	Spv			*spvR* (1)
	CYA		*cyaB* (18)	*cyaA* (2)
	xcp Type II secretion system	*xcpRT* (66)		*xcpQ* (1)

Other virulence factor	RicA	*ricA* (22)

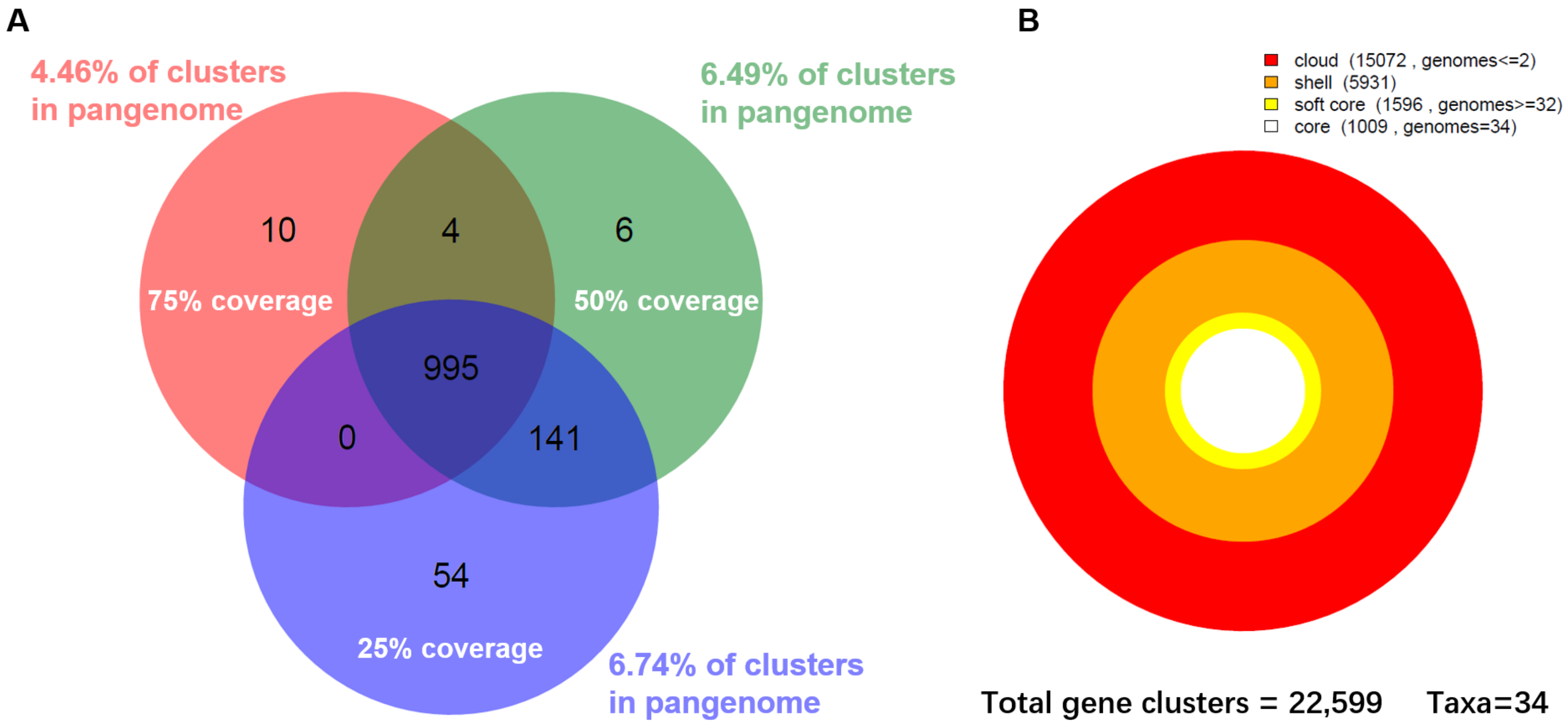

Fig. S1. Venn diagram of pangenomes generated by applying different similarity thresholds (A). Partition of *Comamonas* pangenome into core, soft core, shell and cloud compartments (B).

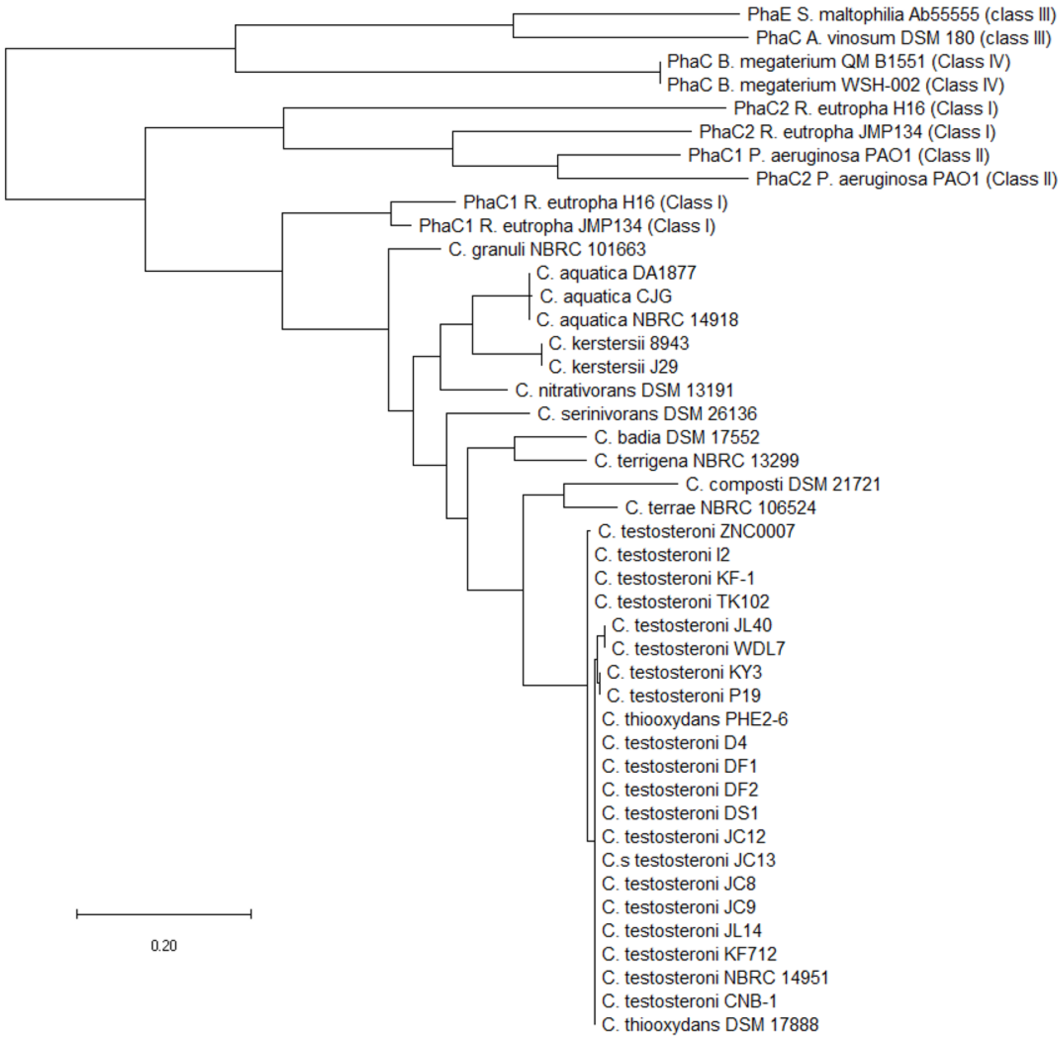

Fig. S2. Maximum likelihood protein sequence phylogenetic analysis based on PhaC of *Comamonas* strains. The scale indicates the number of substitutions per site.

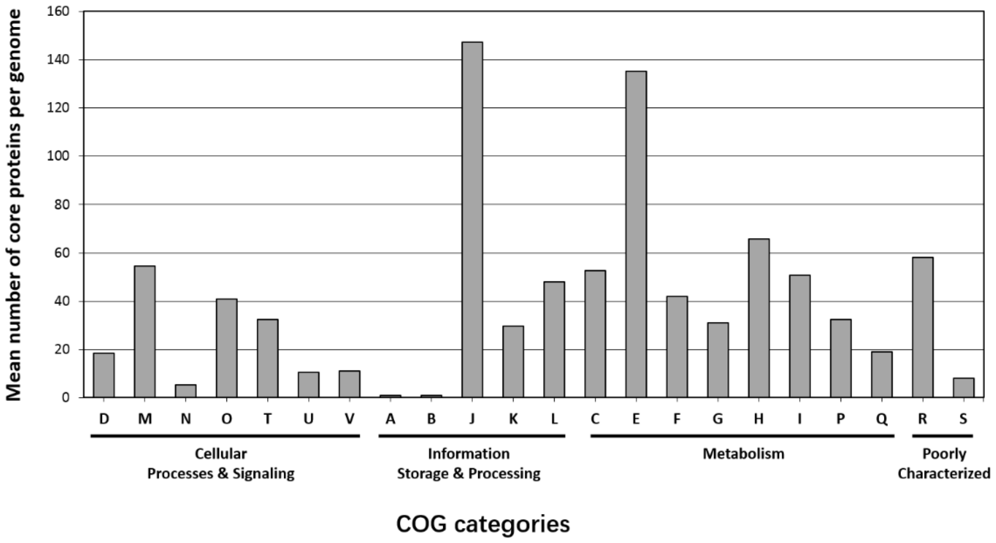

Fig. S3. The average number of core proteins in each genome assigned with COG categories. D, Cell cycle control, cell division, chromosome partitioning; M, Cell wall/membrane/envelope biogenesis; N, Cell motility; O, Posttranslational modification, protein turnover, chaperones; T, Signal transduction mechanisms; U, Intracellular trafficking, secretion, and vesicular transport; V, Defense mechanisms; A, RNA processing and modification; B, Chromatin structure and dynamics; J, Translation, ribosomal structure and biogenesis; K, Transcription; L, Replication, recombination and repair; C, Energy production and conversion; E, Amino acid transport and metabolism; F, Nucleotide transport and metabolism; G, Carbohydrate transport and metabolism; H, Coenzyme transport and metabolism; I, Lipid transport and metabolism; P, Inorganic ion transport and metabolism; Q, Secondary metabolites biosynthesis, transport and catabolism; R, General function prediction only; S, Function unknown.

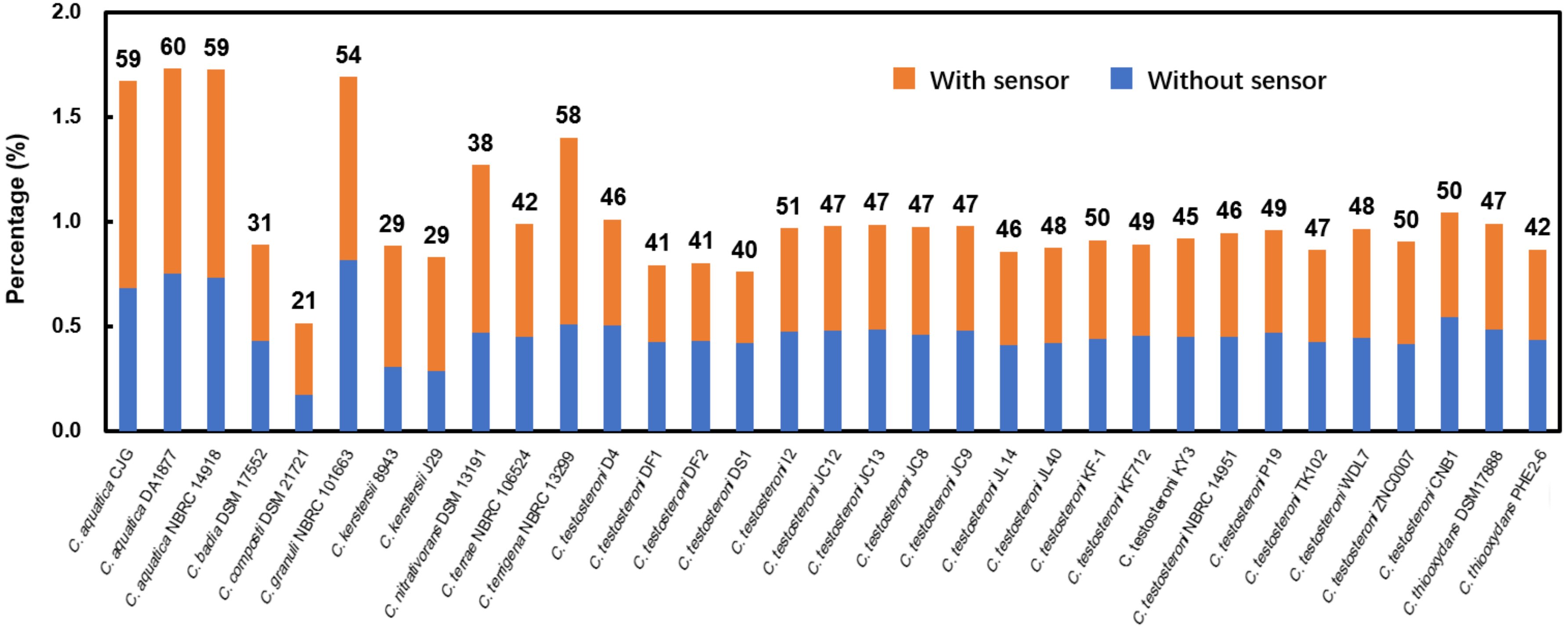

Fig. S4. Percentage of putative c-di-GMP-controlling enzymes in the genome.

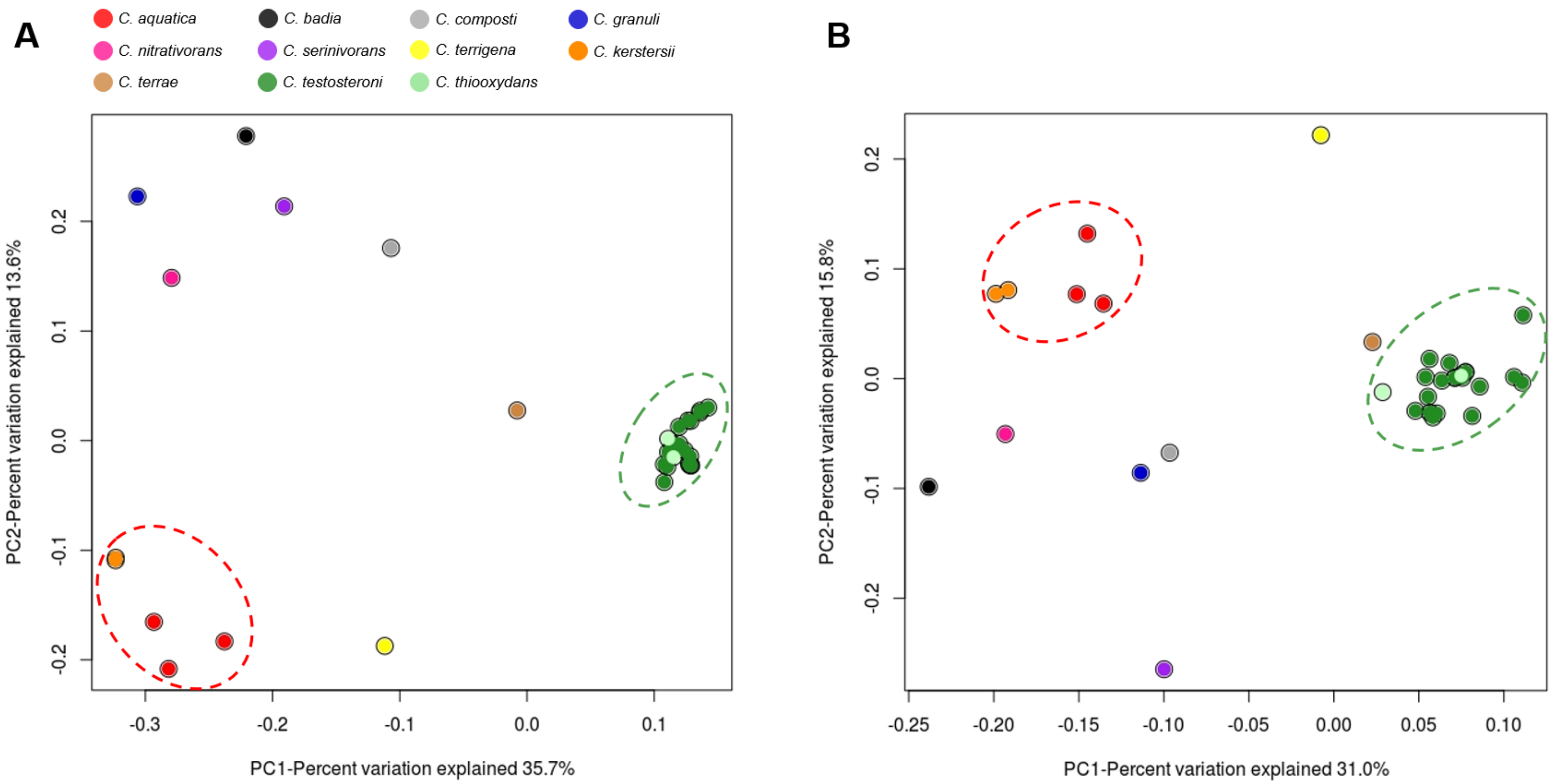

Fig. S5. PCoA plot of virulence factors predicated by MP3 (A) and VRprofile (B).

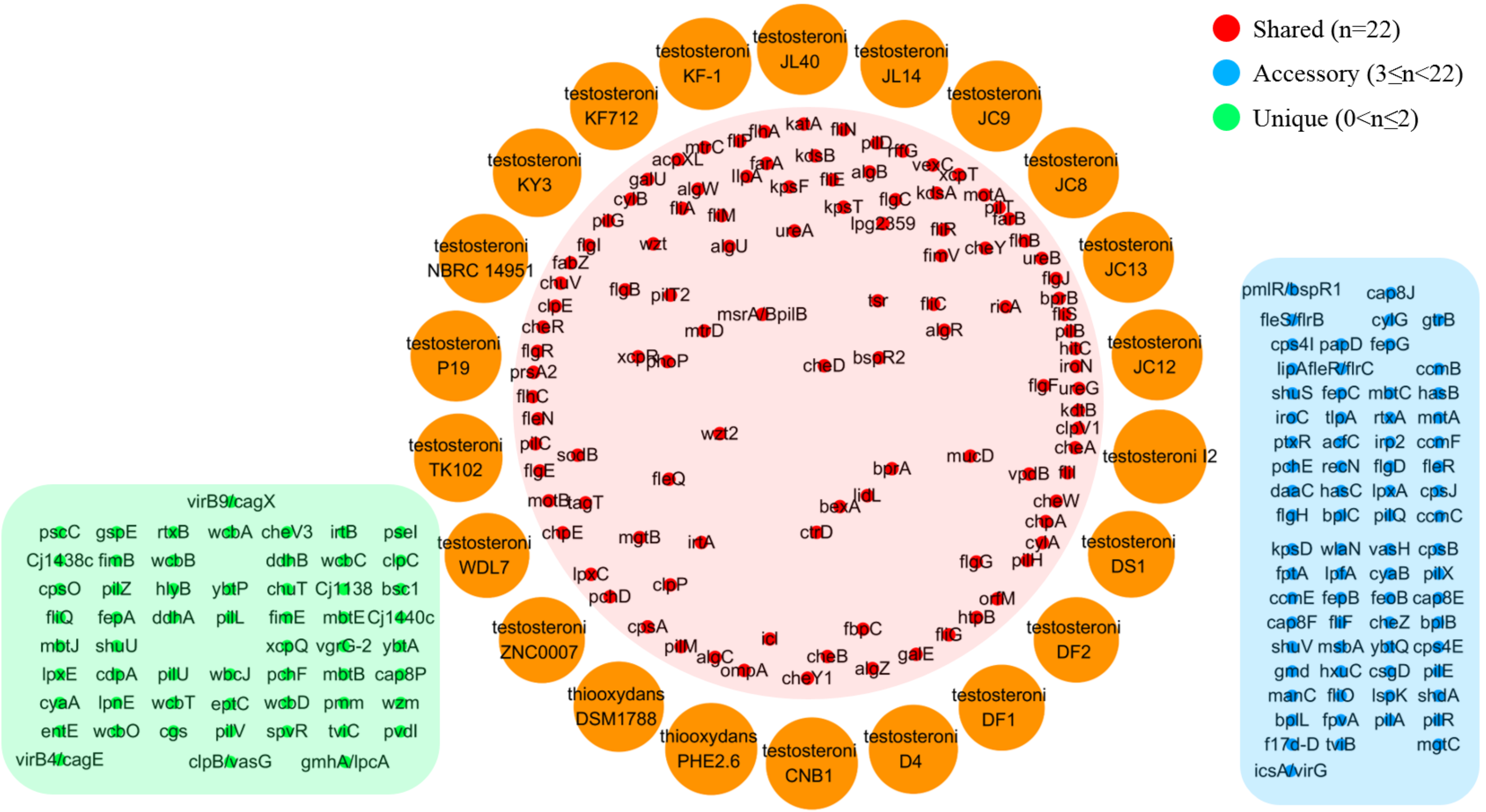

Figure S6. The network analysis reveals the shared (present in all twenty-two strains), accessory (present in more than or equal to three strains) and unique (present in less than or equal to two strains) virulence factors in *C. testosteroni* and *C. thiooxydans*.
